# Autophagy as a targeted therapeutic approach for skin cancer: Evaluating natural and synthetic molecular interventions

**DOI:** 10.1016/j.cpt.2024.01.002

**Published:** 2024-02-01

**Authors:** Md. Liakot Ali, Amdad Hossain Roky, S.M. Asadul Karim Azad, Abdul Halim Shaikat, Jannatul Naima Meem, Emtiajul Hoque, Abu Mohammed Fuad Ahasan, Mohammed Murshedul Islam, Md. Saifur Rahaman Arif, Md. Saqline Mostaq, Md. Zihad Mahmud, Mohammad Nurul Amin, Md. Ashiq Mahmud

**Affiliations:** a Department of Pharmacy, University of Chittagong, Chattogram 4331, Bangladesh; b Pratyasha Health Biomedical Research Center, Dhaka 1230, Bangladesh; c Department of Pharmacy, International Islamic University Chittagong, Chattogram 4318, Bangladesh; d Department of Pharmacy, Daffodil International University, Dhaka 1216, Bangladesh; e Department of Pharmacy, BGC Trust University Bangladesh, Chattogram 4381, Bangladesh; f School of Basic Pharmaceutical and Toxicological Sciences, College of Pharmacy, University of Louisiana at Monroe, Monroe, LA 71209-0497, USA; g Pioneer Dental College and Hospital, Dhaka 1229, Bangladesh

**Keywords:** Autophagy, Skin cancer, Autophagy inducers, Autophagy inhibitors, Nature-derived compounds, Synthetic compounds

## Abstract

Skin cancer, a prevalent malignancy worldwide, poses significant health concerns owing to its increasing incidence. Autophagy, a natural cellular process, is a pivotal event in skin cancer and has advantageous and detrimental effects. This duality has prompted extensive investigations into medical interventions targeting autophagy modulation for their substantial therapeutic potential. This systematic review aimed to investigate the relationship between skin cancer and autophagy and the contribution and mechanism of autophagy modulators in skin cancer. We outlined the effectiveness and safety of targeting autophagy as a promising therapeutic strategy for the treatment of skin cancer. This comprehensive review identified a diverse array of autophagy modulators with promising potential for the treatment of skin cancer. Each of these compounds demonstrates efficacy through distinct physiological mechanisms that have been elucidated in detail. Interestingly, findings from a literature search indicated that none of the natural, synthetic, or semisynthetic compounds exhibited notable adverse effects in either human or animal models. Consequently, this review offers novel mechanistic and therapeutic perspectives on the targeted modulation of autophagy in skin cancer.

## Highlights

• Autophagy-targeted therapies are widely explored for skin cancer due to their great potential.
• Autophagy plays a dual role in skin cancer and is responsible for both cancer progression and inhibition.
• Autophagy modulators, including autophagy inducers and inhibitors from natural, semisynthetic, and synthetic sources, are effective against skin cancer.
• Owing to their safety and effectiveness, autophagy modulators can be a useful addition to treat skin cancer.

## Graphical abstract

**Figure:**
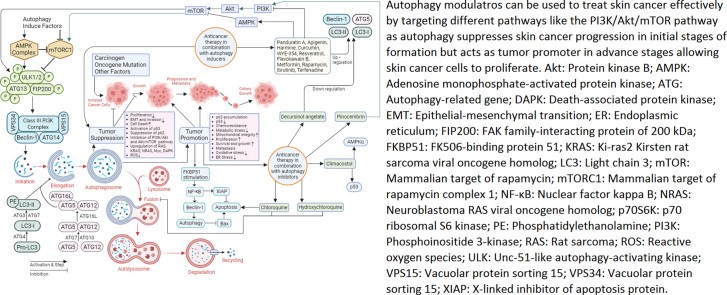

## Introduction

In the United States, skin cancer is a predominant type of cancer, accounting for 40–50 % of all reported cases. It can be broadly categorized into two distinct types: non-melanoma skin cancers (NMSCs), which develop from keratinocytes, and melanoma skin cancers (MSCs), which develop from skin pigment cells called melanocytes. The incidence rate of NMSCs is considerably higher than that of MSCs, but NMSCs have a remarkably low mortality rate. However, MSCs account for approximately 1–4 % of all skin cancer cases and are responsible for approximately 60 % of mortality cases.^1^

The probability of developing skin cancer is determined by a combination of inherent and external factors. Constitutional risk factors, including family history, red hair color, presence of melanocytic nevi, and sensitivity to sun exposure, exert an influence on susceptibility. Additionally, solar ultraviolet (UV) radiation is a widely recognized environmental risk factor for skin cancer.^2^ Surgical excision is the primary treatment for skin cancer, and the decision to undergo this intervention is influenced by factors such as patient health, potential tissue reactions, and ability to tolerate multiple treatment sessions.^3^ Other therapies, such as chemotherapy, immunotherapy, and targeted therapy, are occasionally useful but have certain limitations, such as high cost, increased rate of recurrence, and adverse toxic effects on the patient's body.^1^^,^^4^ Consequently, there is a need to develop new and more efficient methods for the management of skin cancer. Autophagy has recently emerged as a promising pathway that is thought to play a significant role in the progression and treatment of skin cancer.^5^^,^^6^

Autophagy is a conserved, intracellular, self-degradative mechanism that performs several functions in maintaining cellular homeostasis. Cellular stress induced by hunger, organelle destruction, or proteotoxic aggregates enhances autophagy, which harnesses the degradation capabilities of lysosomal enzymes to ameliorate intracellular stress.^7^ Autophagy performs crucial functions in cell survival and maintenance through the degradation of cytoplasmic organelles, proteins, and macromolecules and the recycling of residual products. The malfunction of this mechanism plays a role in the pathology of several human diseases, such as respiratory, neurodegenerative, and cardiovascular diseases, infection, tumors, and cancer.^8^^,^^9^ Owing to the instability of the genome, faulty cell development, and effects of cell stress, chronic suppression or absence of autophagy promotes cancer.^10^ Numerous studies have demonstrated the involvement of autophagy in cancer. However, the precise effect of autophagy on tumor development remains unclear, and its role as either a tumor suppressor or enhancer has not been fully established, which makes its role in cancer treatment controversial. However, the processes through which autophagy operates relate to cancer being involved in several alterations of autophagy-linked proteins, such as autophagy-related genes (ATGs), p53, Beclin 1, mammalian target of rapamycin (mTOR), and Ki-ras2 Kirsten rat sarcoma viral oncogene homolog (KRAS), and autophagy pathways, such as mTOR, phosphoinositide 3-kinase (PI3K), mitogen-activated protein kinase (MAPK), reactive oxygen species (ROS), and nuclear factor kappa B (NF-κB).^11^

Accelerated autophagy has been demonstrated in both NMSCs and MSCs, where it plays a dual role.^12^^,^^13^ For example, during the initial phases of cancer development, it can inhibit tumor growth by removing dysfunctional cell components and potentially harmful substances. This process aids in halting the propagation of damage, including DNA alteration. In contrast, autophagy can assist cancer cells in surviving under stressful circumstances, including nutrition restriction, oxygen deprivation, and chemotherapeutic drug exposure, and facilitate the advancement of skin cancer by enhancing angiogenesis and the production of new blood vessels that deliver nutrition and oxygen to cancerous cells.^14^, ^15^, ^16^, ^17^ Autophagy inducers and inhibitors, collectively known as autophagy modulators, have been used to treat skin cancer and found to be comparatively effective and reliable. Although the sources of autophagy modulators are mainly plant-derived phytochemicals, known as natural compounds, a series of semisynthetic and synthetic autophagy modulators have been shown to possess anti-skin cancer activities in various laboratory experiments.

In this review, we aimed to concentrate on and asses the role of autophagy in skin cancer and its potential as an effective drug target for the treatment of skin cancer. To provide a thorough analysis of the findings on autophagy as a prospective drug target instead of conventional skin cancer treatment, we also shed light on the effects and mechanisms of autophagy modulators in various types of skin cancer.

This review focuses on the descriptive interpretation of skin cancer and autophagy and their relationship in the human body and autophagy modulators in skin cancer. Additionally, the clinical significance of certain compounds has been emphasized.

## Skin cancer and its types

The skin is composed of two primary layers: the outer layer, known as the epidermis, primarily consisting of keratinocytes, and the dermis, which comprises fibroblasts, mast cells, macrophages, and Langerhans cells. Melanocytes reside within the basal layer of the epidermis and play a crucial role in supplying and transferring melanin to keratinocytes, resulting in skin pigmentation and protection against UV damage.^1^

Skin cancer is mainly classified into two types based on the cells from which it arises: MSCs (from melanocytes) and NMSCs (from the epidermis). These two major groups account for >90 % of skin cancer cases, whereas other skin tumor types comprise a remarkably minor portion of skin malignancies. NMSCs are divided into two groups, such as basal cell carcinoma (BSC) and squamous cell carcinoma (SCC). Although the prevalence of MSCs is remarkably lower than that of other cell types, they are responsible for more deaths compared to those with other types of skin cancer.^18^

Melanoma is a fatal and aggressive form of cancer and is the most predominant cancer worldwide, with approximately 325,000 newly reported cases and 57,000 fatalities in 2020.^19^ A buildup of genetic alterations that activate oncogenes, deactivate tumor suppressor genes, and hinder DNA repair occurs when melanocytes are exposed to UV radiation. This mechanism may result in unchecked melanocyte growth and, ultimately, malignancy.^20^

All non-melanoma skin malignancies affecting the skin are described using the universal term NMSCs. The term “NMSCs” originally referred to keratinocyte carcinomas, specifically BCC and SCC, considering they account for 99 % of the tumors in this group, particularly epidemiologically.^21^ SCC is one of the most prevalent solid malignancies and a leading cause of death worldwide. As individuals are increasingly exposed to carcinogens, such as UV radiation from sun exposure, smoking, alcohol intake, and human papillomavirus infection, the incidence rate of SCC is rapidly increasing. SCC develops from the epithelium and can be divided into stratified squamous and non-squamous epithelia, which encompass the epithelia of the skin, esophagus, oral cavity, and airways. Several types of SCCs share histological characteristics, such as the development of keratin pearls, which indicate the presence of squamous differentiation.^22^ BCC is the most prevalent skin neoplasm and is typically associated with chronic and regular sun exposure. It develops from pluripotent cells of the follicular epithelium, which typically carry *p53* gene alterations. Occasionally, BCC is caused by *PTCH1* gene mutation, which results in abnormal initiation of the sonic hedgehog signaling pathway.^18^ BCC is not a fast-growing tumor; however, depending on its subtype, it can be relatively aggressive. It rarely spreads to other organs, and most morbidities are caused by ignored or improperly treated lesions that have recurred. BCC typically affects individuals aged >40 years.^23^

## Basic mechanisms and phases involved in autophagy

**Figure 1:**
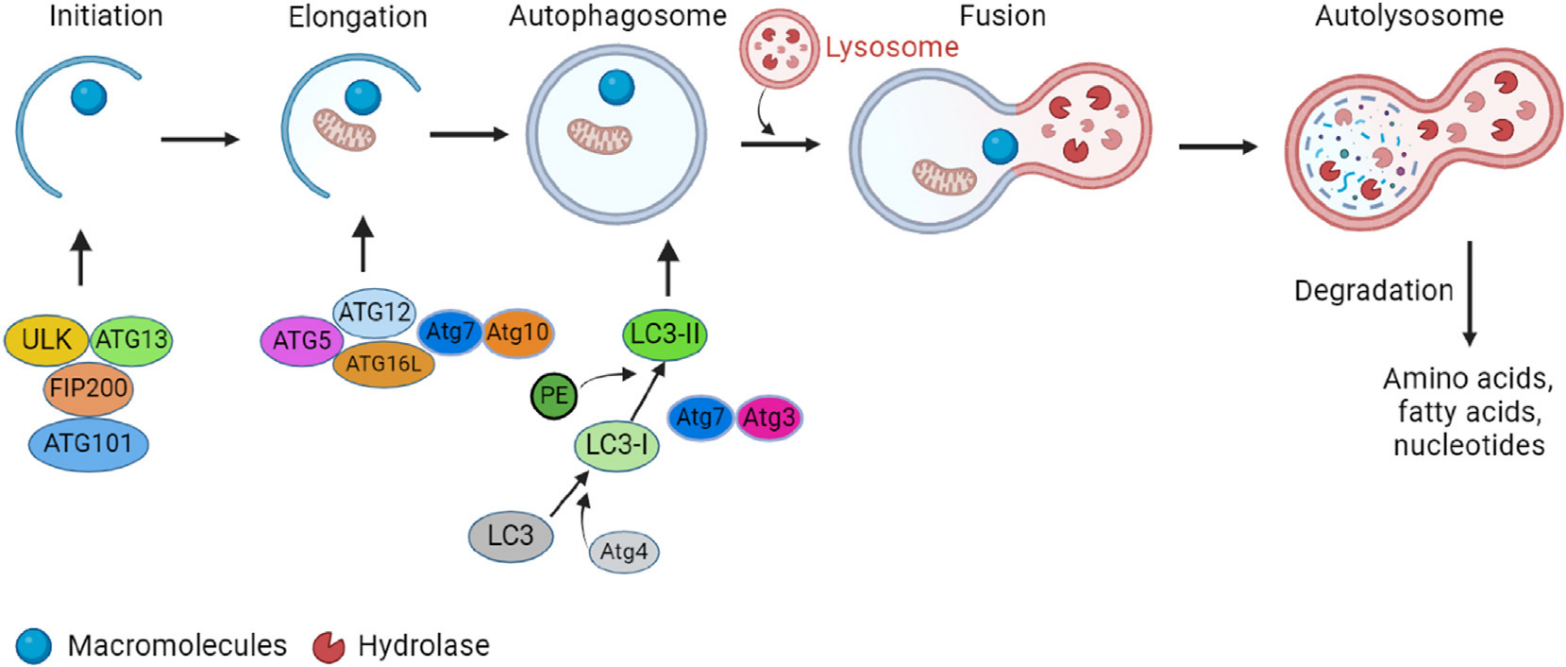
The five stages of autophagy are initiation, elongation and autophagosome formation, fusion, and autolysosome formation. Targeting macromolecules to double-membrane vesicles known as autophagosomes leads to autolysosome formation by lysosome fusion. The Unc-51-like autophagy-activating kinases 1 (ULK1) complex, which includes ULK, autophagy-related gene (ATG) protein 13, FIP200, and ATG101, triggers autophagy. Two ubiquitin-like conjugation systems, such as the ATG12 and microtubule-associated protein 1A/1B-light chain 3 (LC3) systems, are involved in the elongation and maturation of autophagosomes. An autolysosome is formed by the fusion of the autophagosome with the lysosome. This autolysosome breaks down macromolecules into amino acids, fatty acids, and nucleotides.^24^ ATG: Autophagy-related gene; FIP200: FAK family-interacting protein of 200 kDa; LC3: Light chain 3; PE: Phosphatidylethanolamine; ULK: Unc-51-like autophagy-activating kinase; ULK1: Unc-51-like autophagy-activating kinase 1.

Autophagy involves the creation of autophagosomes, which entails enveloping a portion of the cytoplasm along with the organelles and proteins that necessitate degradation within the cells. To maintain cell homeostasis and organelle regeneration, autophagosomes combine with lysosomes to form autophagolysosomes, which break down the contents of the inclusions [Figure 1].^8^ This dynamic process includes autophagosome formation, autophagosome-lysosome fusion, and degradation of intra-autophagosomal contents by lysosomal hydrolases.^25^ The basic mechanism of autophagy is complex. The upstream signaling pathway of autophagy includes the mTOR-dependent and mTOR-independent (adenosine monophosphate-activated protein kinase [AMPK], PI3K, Rat sarcoma (Ras)-MAPK, p53, phosphatase and tensin homolog deleted on chromosome 10 [PTEN], endoplasmic reticulum [ER] stress) pathways.^8^ The PI3-binding proteins, PI3 phosphatases, Rab proteins, the ATG/Unc-51-like autophagy-activating kinase 1 (ULK1) protein-kinase complex, ATG9. ATG2-ATG18 complex, Vps34-ATG6/Beclin 1 class III PI3-kinase complex, and ATG12 and ATG8/light chain 3 (LC3) conjugation systems are some of the proteins and multimolecular complexes that aid the formation of autophagosomes. ATG12 and LC3 conjugations and two ubiquitin-like alterations are necessary for membrane elongation and autophagosome formation.^25^

Autophagy involves five distinct stages: initiation, elongation, maturation, fusion, and degradation. Initially, macromolecules and organelles form cargo and are enclosed by a double-membrane vesicle that slowly expands, finally forming an autophagosome [Figure 1]. Subsequently, the autophagosome fuses with a lysosome, resulting in the emergence of an autolysosome. Within the autolysosome, lysosomal hydrolases break down the cargo, whereas lysosomal permeases recycle the resulting products back into the cytoplasm. The ULK complex, which comprises ULK1/2, ATG13, FAK family-interacting protein of 200 kDa (FIP200), and ATG101, plays a crucial role in the initiation of autophagy. Another key complex required for autophagosomal nucleation is the Beclin-Vps34-ATGq4L-p150 complex.^26^

## Dual role of autophagy in skin cancer

**Figure 2:**
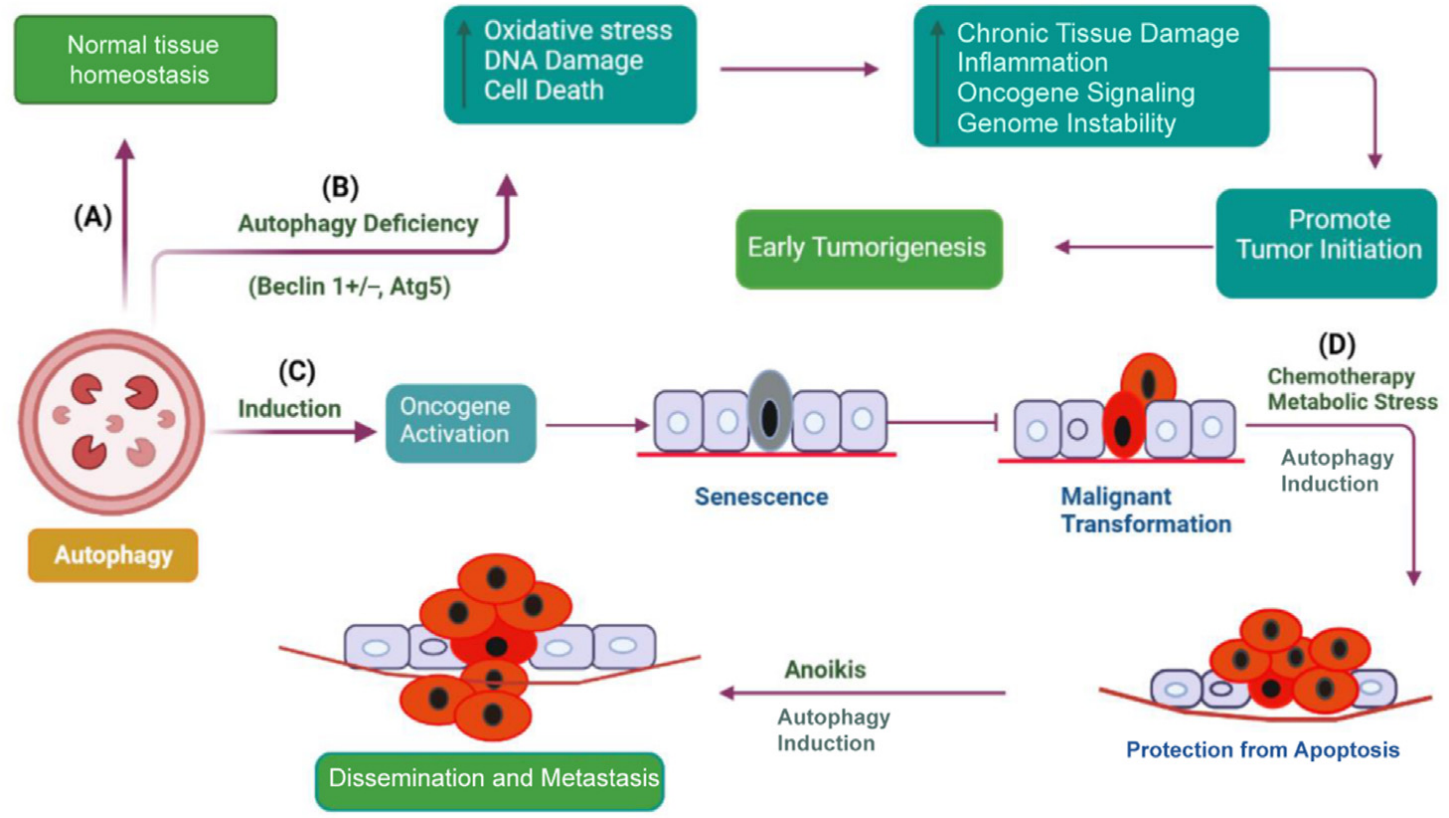
Multifaceted role of autophagy in cancer progression. (A) Autophagy conducts homeostatic functions in normal tissue, such as monitoring the functionality of organelles and proteins. (B) When autophagy is inhibited in tissues, normal homeostasis is disrupted, which increases inflammation, reactive oxygen species (ROS) levels, and DNA damage (genomic instability and aneuploidy). Taken together, these modifications may encourage the development of tumors and trigger early tumorigenesis.^16^ (C) Autophagy accelerates oncogene-induced senescence, which inhibits malignant transformation. (D) After several stimuli, such as chemotherapy, metabolic stress, and anoikis, autophagy induction increases tumor cell survival, which may encourage drug resistance and metastasis.^27^ ROS: reactive oxygen species.

Autophagy has a complex role in cancer [Figure 2] and is influenced by cancer type, stage, genetic background, and tumor microenvironment.^28^ Autophagy generally plays a tumor-suppressive role in normal cells and benign tumors, but it also helps established cancers survive and become treatment-resistant.^29^^,^^30^ Therefore, the role of autophagy in skin cancer remains controversial. Autophagy acts as a tumor suppressor by reducing the buildup of damaged organelles and regulating cell proliferation and genetic instability.^31^ Beclin 1^+/−^ mice have increased susceptibility to malignancies^32^ and show improved angiogenesis, development, and migration of implanted melanoma tumors. Allelic^33^ loss of the crucial autophagy gene Beclin 1 has been found in 40–75 % of human prostate, breast, and ovarian cancers,^34^^,^^35^ indicating a function for autophagy in tumor suppression. Ironically, autophagy may also aid in the growth of preexisting malignancies by assisting with cellular metabolic demands.^36^ Knockdown of essential ATGs in tumor cells may enhance the induction of apoptosis.^37^

In 2013, Liu et al^38^ demonstrated that during the early stages of malignant transformation in skin cancers, such as melanoma, autophagy was diminished compared with that in normal melanocytes, correlating with downregulated ATG5. Tumor ATG5 expression was lower than that in benign nevi and normal skin cells. Their study revealed a significant association between ATG5 expression levels in melanoma samples from patients and progression-free survival, indicating that higher ATG5 levels in tumors were associated with a more favorable outcome.

Similarly, increasing ATG5 levels in cultivated tumor cells prevent their growth and lead to senescence.^38^ Expression of other autophagic markers, such as LC3 and Beclin 1, is also reduced in abnormal melanocytic growth, in addition to ATG5. This indicates decreased autophagy during the early stages of melanoma formation. This phenomenon was also observed by Miracco et al^39^ in 2010 and Hara and Nakamura^40^ in 2012. Furthermore, Maes et al^41^ reported that autophagic flux was significantly reduced in the early stages of melanoma development, with increased protein kinase B (Akt) activity. Unexpectedly, as melanoma progresses, metastatic melanoma cells regain their capacity to trigger autophagic flow, thereby promoting survival.^41^ This indicates that autophagy plays a tumor-suppressive role in melanocytes during the initial stages of skin cancer formation.

In contrast, skin cancer cells in advanced stages exhibit relatively higher overexpression of Beclin 1 and LC3, indicating higher autophagy compared to that in the initial primary lesions.^42^ Thus, mature skin cancers with elevated autophagy levels, such as melanoma and SCC, are associated with tumor aggressiveness.^43^, ^44^, ^45^, ^46^ Established melanoma cells employ autophagy as a protective mechanism to survive in harsh environments. Thus, increased autophagy aids melanoma cell survival and promotes their growth, directly contributing to cancer progression.^42^ In 2012, Marino et al^47^ found that melanoma cells, when cultured under acidic conditions, could enhance their survival by elevating autophagic activity. The survival of melanoma cells in acidic environments was reduced when autophagy was inhibited by ATG5 silencing.^47^ According to a recent study, the flavonoid luteolin can enhance the cell death caused by chloroquine by inhibiting autophagy in metastatic SCC cells. These findings suggest that the upregulation of autophagy acts as a cytoprotective mechanism in squamous cell cancer.^48^ Therefore, melanomas with high autophagy levels are more likely to withstand treatment than melanomas with low autophagy levels. According to a previous study, radiation stimulated the expression of FK506-binding protein 51 (FKBP51) in malignant melanomas. FKBP51 enhances NF-κB activation, which has two effects: it increases Beclin 1 expression, which triggers autophagy, and it stimulates X-linked inhibitor of apoptosis protein (XIAP), which suppresses apoptosis. Bax is then degraded via autophagy, preventing apoptosis. If FKBP51 treatment is stopped, cells undergo apoptosis, rather than NF-κB activation following radiation therapy.^49^

From the above discussion, it can be concluded that autophagy plays a tumor-suppressive role in the early stages of skin cancer formation and a tumor-promoting role in the advanced stages of skin cancer. However, how and why autophagy changes its role throughout the process remains controversial, although the autophagic process uses the same cellular machinery in both early and advanced cases of skin cancer. However, because both the induction and inhibition of autophagy are closely related to skin cancer progression and prevention, autophagy can be used as a potential therapeutic target to more effectively treat skin cancer.

## Autophagy modulators against skin cancer

**Figure 3:**
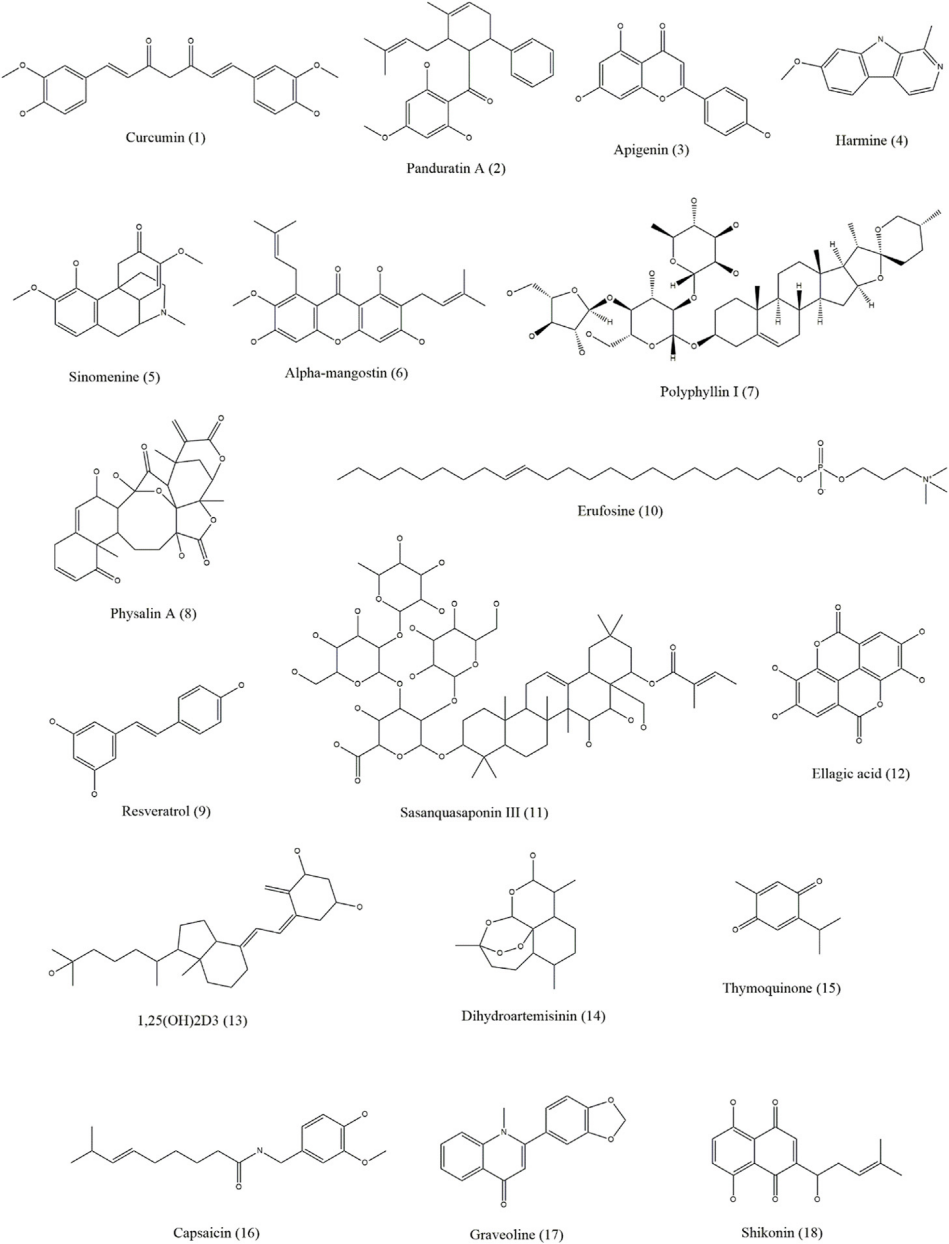
Chemical structures of compounds (1–18) that show efficacy against skin cancer by influencing autophagy.

**Figure 4:**
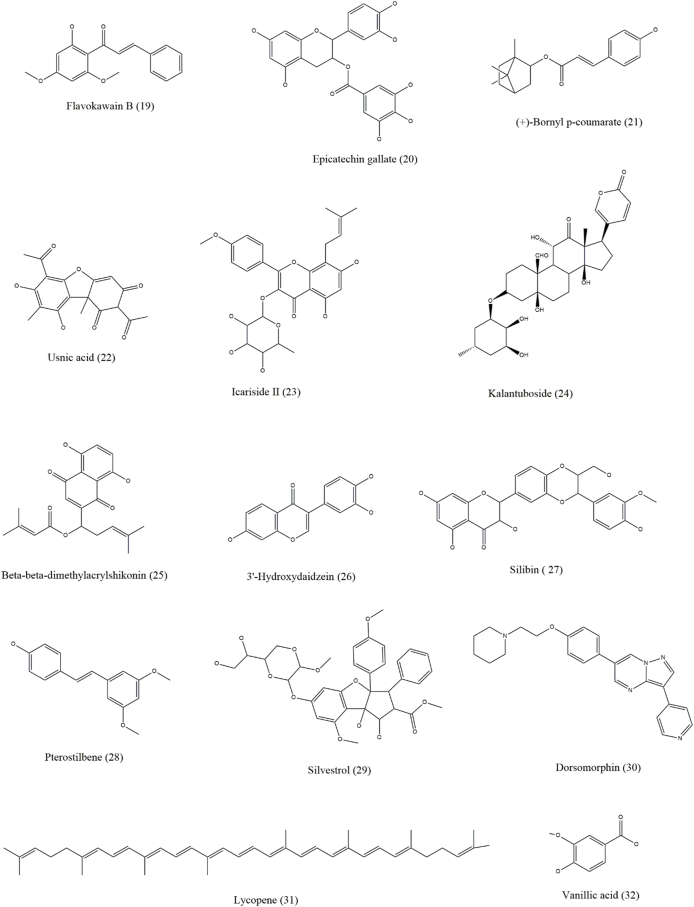
Chemical structures of compounds (19–32) that show efficacy against skin cancer by influencing autophagy.

**Figure 5:**
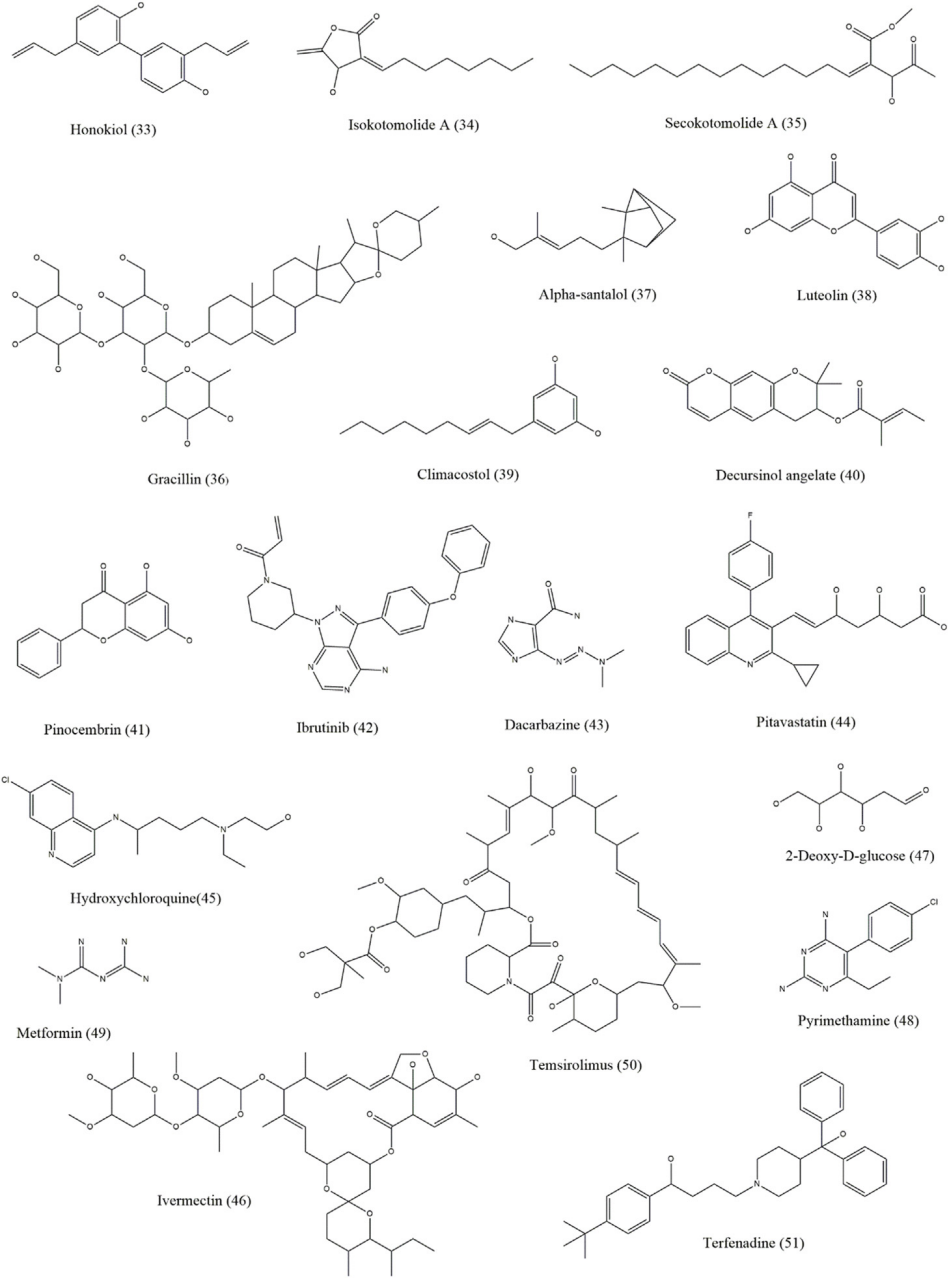
Chemical structures of compounds (33–51) that show efficacy against skin cancer by influencing autophagy.

**Figure 6:**
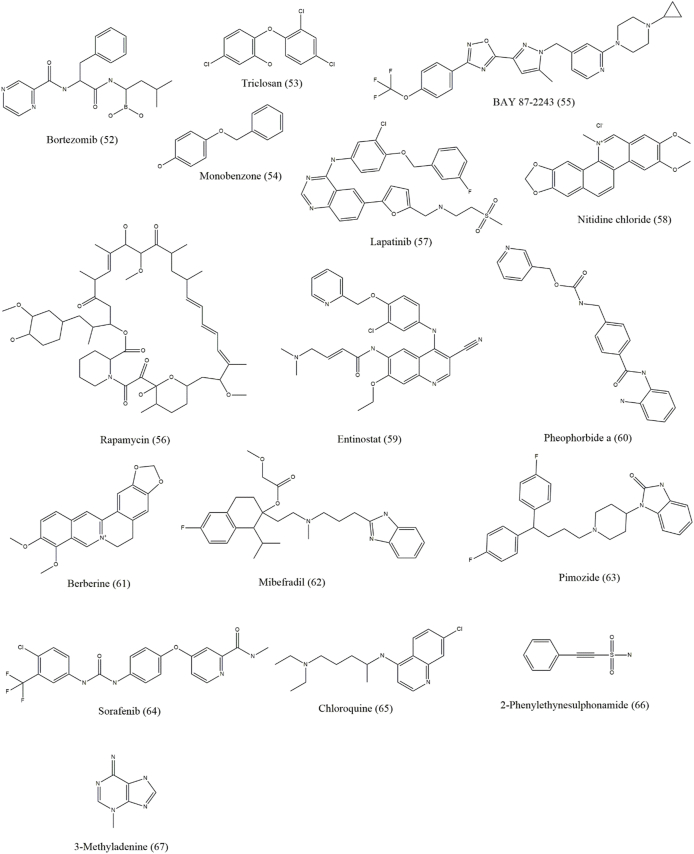
Chemical structures of compounds (53–67) that show efficacy against skin cancer by influencing autophagy.

Both autophagy inhibitors and inducers, collectively known as autophagy modulators, are useful against skin cancer [Figure 3, Figure 4, Figure 5, Figure 6 and Table 1]. As autophagy in skin cancer aids tumor cell survival in an unfavorable environment, the inhibition of autophagy may be a useful treatment approach for skin cancer that has progressed to an advanced stage. Notably, the use of autophagy inhibitors can potentially induce localized inflammation, which in turn may promote immunogenic cell death and provide tumor-resistant cells the option for apoptosis. In contrast to its function as a defense against chemotherapy, it may also be associated with drug-induced cytotoxicity in malignancies. Several medications with the ability to kill malignant cells may also activate autophagy.^1^

**Table 1:** Role and mechanism of autophagy modulators in various types of skin cancer.

Compound name	Mechanism of action	Skin cancer types	References
Curcumin (diferuloylmethane)	Downregulation of the Akt/mTOR signaling pathway and ROS generation and upregulation of Beclin 1	Melanoma	50
Panduratin A	Downregulation of the mTOR pathway and activation of the AMPK pathway	Melanoma	51
Apigenin	Downregulation of the mTOR pathway and activation of the AMPK pathway	Keratinocyte carcinoma	52
Sinomenine	Inhibition of the PI3K/Akt/mTOR pathway and increased expression of Beclin 1 and LC3-II/LC3-I	Melanoma	53
α-Mangostin	Inhibition of the PI3K/Akt/mTOR pathway and increased expression of Beclin 1, LC3-I, and LC3-II	Melanoma	54
Polyphyllin I	Inhibition of the PI3K/Akt/mTOR pathway and increased expression of Beclin 1, LC3, and LC3-II	Melanoma	55
Physalin A	Upregulation of the p38-NF-κB pathway, increased expression of Beclin 1, and regulation of LC3-II/LC3-I	Melanoma	56
Resveratrol	Modulation of Rictor and suppression of the Akt/mTOR pathway	Squamous cell carcinoma and melanoma	57,58
Erufosine	Inhibition of the Akt/mTOR pathway	Squamous cell carcinoma	59
Harmine	Suppression of the Akt/mTOR pathway	Melanoma	60
Sasanquasaponin III	Inhibition of the Akt/mTOR/p70S6K pathway, ROS accumulation, and upregulation of LC3-II expression	Melanoma	61
Ellagic acid	Inhibition of the PI3K/Akt/mTOR pathway	Melanoma	62
Dihydroartemisinin	Increased accumulation of autophagosomes	Tongue squamous cell carcinoma	63
Thymoquinone	Increased accumulation of autophagosomes	Head and neck squamous cell carcinoma	64
Protoapigenone	ROS generation and increased p53 and Beclin 1 expression	Melanoma	65
Graveoline	ROS generation and increased p53 and Beclin 1 expression	Melanoma	66
Shikonin	ROS generation, activation of the p38 pathway, and increased p-p38, LC3B-II, and Beclin 1 expression	Melanoma	67
Flavokawain B	ROS generation, mTOR inhibition, and increased LC3-II levels	Melanoma	68
Novel ECG analog 4-(S)-(2,4,6-trimethylthiobenzyl)-epigallocatechin gallate	ROS accumulation	Melanoma	69
Usnic acid	ROS generation	Melanoma	70
Icariside II	ROS generation and inhibition of the MITF, JAK-STAT3, and MAPK pathways	Melanoma	71,72
Kalantuboside B	ROS generation, p53 downregulation, and LC3-II accumulation	Melanoma	73
β-β-Dimethylacrylshikonin	ROS generation and increased LC3B-II expression	Melanoma	74
3′-Hydroxydaidzein	Up-regulation of ATG5	Melanoma	75
Luteolin	Activation of acidic lysosomal vacuolization and accumulation of LC3-II	Squamous cell carcinoma	75
Silibinin	Inhibition of the IGF-1R pathway and increased Beclin 1 and ATG5 expression	Epidermoid carcinoma	76
Pterostilbene	Activation of ULK1 and ATG13 by activating the AMPK pathway	Melanoma	77
Silvestrol	Autophagosome formation and accumulation of LC3-II	Melanoma	78
Dorsomorphin	ROS generation and LC3-II expression	Basal cell carcinoma	79
*Polygonatum cyrtonema* lectin	Regulation of the ROS-p38-p53 pathway	Melanoma	80
Capsaicin	Increasing ULK1 acetylation and promotion of ROS-dependent autophagy	Melanoma	81
(+)-Bornyl p-coumarate	Upregulation of the expression of Beclin 1, ATG3, ATG5, p62, LC3-I, and LC3-II proteins	Melanoma	82
1,25(OH)2D3	Increased DDIT4 expression and inhibition of mTORC1 activity to promote autophagy	Squamous cell carcinoma	83
Lycopene	Increased levels of the autophagy protein p62, causing Keap1 to be degraded through an autophagy-lysosomal pathway	Cutaneous tumor	84
Vanillic acid	Induction of STAT3-mediated autophagy	Melanoma	85
Honokiol	Activation of LC3B	Melanoma	86
Nitidine chloride	Inhibition of the mTOR pathway	Melanoma	87
Isokotomolide A	Formation of autophagic *vacuoles*	Melanoma	88
Secokotomolide A	Formation of autophagic vacuoles	Melanoma	88
Gracillin	Suppression of phosphorylation/activation of PI3K, Akt, and mTOR	Melanoma	89
Hibiscus leaf polyphenolic (HLP) extract	Increased expression of ATG5, Beclin 1, and LC3-II	Melanoma	90
Diethyl ether (Et_2_O) fraction of *Jasione montana* extract	Induction of autophagy	Melanoma	91
Natto	Formation of acidic autophagic vacuoles and autophagolysosomes	Melanoma	92
East Indian sandalwood oil	Activation of LC3B	Skin cancer	93
Chinese propolis	Decreased ratio of LC3-I/LC3-II and amplification of ATG5/ATG12 complex and p62 protein	Melanoma	94
Ibrutinib	Induction of autophagy and inhibition of cell proliferation by concentration and time-dependent manner	Skin cancer	95
Novel binuclear palladium metallacycle complex with 1,2-bis(dipenylphosphino)ethane	Increased levels of LC3-II and Beclin 1	Melanoma	96
Terfenadine	Promotion of autophagy in both ROS-independent and ROS-dependent manner	Melanoma	97
Bortezomib	Breakdown of LC3 and autophagic formation	Melanoma	98
Triclosan	Through the AMPK/p62/LC3 pathway	Melanoma	99
Neratinib and entinostat combination	ROS-dependent activation of ATM via AMPK-ULK1-ATG13-Beclin 1/ATG5	Melanoma	100
Monobenzone	Induction of tyrosinase ubiquitination and the autophagocytic degradation of melanosomes	Melanoma	101
BAY 87-2243	Promotion of autophagosome formation and mitophagy	Melanoma	102
HA15	Increased ER stress, causing the induction of autophagy	Melanoma	103
Flavonoid GL-V9	Blockage of the Akt/mTOR pathway	Squamous cell carcinoma	104
Lapatinib	Inhibition of the PI3K/Akt/mTOR signaling pathway	Squamous cell carcinoma	105
Temozolomide	Induction of autophagy	Melanoma	1
Dacarbazine	Induction of autophagy	Melanoma	1
Dacarbazine and pitavastatin combination	Increased LC3-II expression	Melanoma	106
Ag/ZnO nanoparticles	Induction of autophagy in response to oxidative stress caused by nanoparticles	Melanoma	107
Nitrogen-doped titanium dioxide nanoparticles	Induction of autophagy in the dark and inhibiting autophagy in the light	Melanoma	108
Nitrogen-phosphorous-doped carbon dot	Upregulation of the protein expression levels of LC3-II and ATG-5	Melanoma	109
Pheophorbide a	Increased expression of the ATGs Beclin 1, LC3B, and ATG5	Melanoma	110
Berberine	Increased level of LC3-related autophagy	Melanoma	111
2-Deoxy-d-glucose	Mitochondria hyperpolarization	Melanoma	112
Pyrimethamine	Increased autophagosome and LC3-II expression	Melanoma	113
Metformin	Regulation of LC3 and Beclin 1 proteins	Melanoma	114
Ivermectin	ROS generation	Melanoma	115
AC-1001-H3	ROS generation and increased LC3/LC3-II and Beclin 1 expression	Melanoma	116
Pyr-1	Enhanced Akt-dependent autophagy	Melanoma	117
Protoapigenone 1′-O-butyl ether	ROS generation	Melanoma	65
Rapamycin	Inhibition of the mTOR pathway and increased Bcl-2, Bax, and LC3-II expressions	Melanoma	118
Hydroxychloroquine	Inhibition of autophagy by preventing the fusion of autophagosomes with lysosomes	Melanoma	119
Hydroxychloroquine and temsirolimus	Concomitant inhibition of mTOR and autophagy	Melanoma	106
Climacostol	Inhibition of autophagy by increasing p53 protein levels and activating AMPKα	Melanoma	120
Decursinol angelate	Inhibition of autophagy by decreasing ATG5, ATG7, Beclin 1, and LC3-II expressions	Melanoma	121
Pinocembrin (5,7-dihydroxy flavanone)	Inhibition of autophagy via activating the PI3K/Akt/mTOR pathway	Melanoma	122
Chloroquine	Modification of mTOR-mediated autophagy, causing the inhibition of autophagy	Melanoma	123
Bafilomycin A1 and ammonium chloride combination	Activation of the p38 MAPK and autophagy inhibition	Melanoma	124
3-Methyladenine and 5-fluorouracil	Inhibition of the *LC3* gene in cancer cells	Squamous cell carcinoma	125
Analog of photoactive NADPH NS1	Selective modification of autophagosome formation and autophagy inhibition at the late stage	Melanoma	126
Esomeprazole	Increased accumulation of LC3-II and autophagy inhibition	Melanoma	127
Mibefradil and pimozide	Increased LC3-II/I ratio, accumulation of p62/SQSTM1, and autophagy inhibition	Melanoma	128
Sorafenib	Inhibition of autophagy	Melanoma	1
Sorafenib + α-mangostin	α-Mangostin enhances the activity of sorafenib	NRAS-mutant melanoma	129

Akt: Protein kinase B; AMPK: Adenosine monophosphate-activated protein kinase; ATG: Autophagy-related gene; ATM: Ataxia telangiectasia mutated; DDIT4: Deoxyribonucleic acid damage-inducible transcript 4; ECG: Epicatechin gallate; ER: Endoplasmic reticulum; Et_2_O: Diethyl ether; HLP: Hibiscus leaf polyphenolic; IGF-1R: Insulin-like growth factor 1 receptor; JAK: Janus kinase; LC3: Light chain 3; MAPK: Mitogen-activated protein kinase; MITF: Microphthalmia-associated transcription factor; mTOR: Mammalian target of rapamycin; mTORC1: Mammalian target of rapamycin complex 1; NADPH: Nicotinamide adenine dinucleotide phosphate; NF-κB: Nuclear factor kappa B; NRAS: Neuroblastoma RAS viral oncogene homolog; p70S6K: p70 ribosomal S6 kinase; PI3K: Phosphoinositide 3-kinase; ROS: Reactive oxygen species; STAT3: Signal transducer and activator of transcription 3; ULK1: Unc-51-like autophagy-activating kinase 1.

### Natural compounds and extracts inducing autophagy in skin cancer

Panduratin A, apigenin, harmine, sinomenine, curcumin, resveratrol, erufosine, WYE-354, and α-mangostin are some of the natural compounds involved in inducing autophagy in skin cancer by targeting the PI3K/Akt/mTOR pathway. Curcumin (diferuloylmethane) is an active polyphenolic compound found in the rhizome of *Curcuma longa* that induces autophagy in human melanoma cells by downregulating the Akt/mTOR signaling pathway and ROS generation and upregulating Beclin 1.^50^ Panduratin A is a natural chalcone obtained from the rhizome of *Boesenbergia rotunda* that induces autophagy by downregulating the mTOR pathway and activating the AMPK pathway in MSC.^51^ This mechanism has also been observed for apigenin, which induces autophagy in keratinocytes, including basal and squamous cells.^52^ Harmine is an alkaloid found in *Peganum harmala* seeds that suppresses the Akt/mTOR pathway to activate autophagy in B16 cells and is a possible therapeutic agent for MSC.^60^ Sinomenine, which is a plant alkaloid obtained from the root of *Sinomenium acutum*, enhances autophagy in B16 melanoma cells by inhibiting the PI3K/Akt/mTOR pathway and increasing the expression of Beclin 1 and LC3-II/LC3-I.^53^ Inhibition of the PI3K/Akt/mTOR pathway to enhance autophagy and overexpression of Beclin 1, LC3, and LC3-II are also observed in α-mangostin and polyphyllin I in melanoma cells.^54^^,^^55^ Physalin A, a steroidal compound of physalis plants, induces autophagy in melanoma A375-S2 cells by upregulating the p38-NF-κB pathway and activating the expression of Beclin 1 and regulating LC3-II/LC3-I proportions.^56^ Resveratrol (*trans*-3,5,4′-trihydroxystilbene), which is a stilbenoid phenolic compound found in berries, grapes, and peanuts, induces autophagy by modulating Rictor (a component of mammalian target of rapamycin complex [mTORC]2) in squamous carcinoma cells^57^ and activates autophagy in B16 melanoma cells by accumulating ceramide and suppressing the Akt/mTOR pathway.^58^ Erucylphospho-*N*,*N,N*-trimethylpropylammonium (Erufosine) belongs to the alkylphosphocholine class and enhances autophagy in SCC by inhibiting the Akt/mTOR pathway.^59^ Sasanquasaponin III is a triterpenoid saponin obtained from *Schima crenata Korth* that induces autophagy in melanoma A375 cells by Akt/mTOR/p70 ribosomal S6 kinase (p70S6K) pathway inhibition, ROS accumulation, and LC3-II expression upregulation.^61^ Ellagic acid, a polyphenol obtained from the hydrolysis of ellagitannins, induces autophagy in melanocytes by downregulating the phosphorylation of PI3K/Akt and the mTOR pathway.^62^ An active form of vitamin D, 1,25(OH)2D3 has an important protective role against SCC through autophagy. Treatment with 1,25(OH)2D3 increases DNA damage-inducible transcript 4 (DDIT4) expression, inhibits mTORC1 activity to promote autophagy and induces an antiproliferative reaction in SCC cells. Additionally, according to a previous study, after receiving 1,25(OH)2D3, there was a notable reduction in the size and expansion of the tumor, and the tumor-suppressive effects were dependent on vitamin D-DDIT4 interaction.^83^ Dihydroartemisinin, which is an active metabolite of artemisinin isolated from *Artemisia annua*, and thymoquinone obtained from *Nigella sativa* show increased accumulation of autophagosomes and induction of autophagy in human tongue SSC (with increased Beclin 1 and LC3B-II levels) and head and neck SCC (with increased LC3-II protein levels), respectively.^63^^,^^64^

Some compounds, such as capsaicin, protoapigenone, shikonin, graveoline, flavokawain B, epigallocatechin gallate (EGCG), epicatechin gallate (ECG), usnic acid, icariside II, and kalantuboside B, induce autophagy in skin cancer by regulating and accumulating ROS or by increasing the expression of autophagy-related proteins. Capsaicin is a bioactive compound found in chili peppers and is derived from plants of the genus *Capsicum*, the most widely used chili worldwide. Capsaicin directly inhibits cellular tumor-associated nicotinamide adenine dinucleotide (NADH) oxidase (tNOX), leading to a concurrent decrease in silent mating type information regulation 2 homolog 1 (SIRT1) expression. In melanoma cells, this results in increased ULK1 acetylation, which promotes ROS-dependent autophagy. Furthermore, in an *in vivo* xenograft study employing tNOX-depleted melanoma cells, capsaicin therapy effectively suppressed tumor expansion by reducing tNOX and SIRT1 expression.^81^ Graveoline (obtained from *Ruta graveolens*) stimulates ROS generation, is cytotoxic in melanoma A375 cells, and induces autophagy by upregulating p53 and Beclin 1 expression.^66^ Additionally, autophagy in melanoma A375 cells is induced by shikonin, a naphthoquinone isolated from *Lithospermum erythrorhizon*, and is involved in ROS-mediated ER stress, activation of p38 pathways, and upregulation of p-p38, LC3B-II, and Beclin 1 expression.^67^ Flavokawain B, a chalcone, induces ROS-mediated autophagy in melanoma A375 cells by inhibiting mTOR, increasing LC3-II levels, and dysregulating Beclin 1/Bcl-2 levels.^68^ Moreover, ECG and other polyphenols found at high concentrations in hibiscus leaf polyphenolic (HLP) extract cause human melanoma cell death and autophagy. In A375 cells, HLP and ECG cause the cleavage of caspases, regulation of Bcl-2 family proteins, and activation of Fas/FasL. HLP may increase the expression of ATG5, Beclin 1, LC3-II, and autophagy-related proteins and cause autophagic cell death in A375 cells.^90^ (+)-Bornyl p-coumarate is a bioactive substance abundantly found in *Piper betle* stem. It induces autophagy in melanoma cells, as evidenced by the upregulated expression of Beclin 1, ATG3, ATG5, p62, LC3-I, and LC3-II proteins and the inhibition of these proteins by autophagy, preventing 3-methyladenine (3-MA).^82^ Usnic acid, a supercritical CO_2_ extract of *Usnea barbata*, exhibits cytotoxic activity, upregulates ROS production in melanoma B16 cells, and induces autophagy.^70^ Icariside II is a flavonoid extracted from *Herba Epimedii* that induces autophagy in melanocytes by activating ROS production and inhibiting the microphthalmia-associated transcription factor (MITF), Janus kinase (JAK)-signal transducer and activator of transcription 3 (STAT3), and MAPK pathways.^71^^,^^72^ Kalantuboside B is a bufadienolide obtained from *Kalanchoe tubiflora* that induces autophagy in melanoma A2058 cells through ROS generation, p53 downregulation, extracellular signal-regulated kinase (ERK) pathway upregulation, and LC3-II accumulation.^73^ β-β-Dimethyl acryl shikonin, obtained from the roots of *Onosma paniculata*, induces autophagy in melanoma cell lines by ROS generation and increases LC3B-II expression.^74^ 3′-Hydroxydaidzein is an ortho-dihydroxyisoflavone that upregulates ATG5 expression to enhance autophagy in melanoma. Luteolin is a flavone that induces autophagy by initiating intracellular acidic lysosomal vacuolization and buildup of LC3-II in SCC.^75^ Silibinin, a flavonolignan, enhances autophagy in carcinoma A431 cells by inhibiting the insulin-like growth factor 1 receptor (IGF-1R) pathway and upregulating Beclin 1 and ATG5 expression.^76^ Pterostilbene, an analog of resveratrol, induces autophagy through the activation of ULK1 and ATG13 via the AMPK pathway.^77^ Silvestrol, obtained from the fruits and twigs of *Aglaia foveolata*, activates early autophagy by autophagosome formation and the buildup of LC3-II in melanoma cells.^78^ Dorsomorphin, an AMPK inhibitor, induces autophagy in BSC via ROS generation and conversion of LC3-I to LC3-II.^79^ *Polygonatum cyrtonema* lectin (mannose/sialic acid-binding lectin), found in the rhizomes of *P. cyrtonema Hua*, induces autophagy in melanoma A375 cells by regulating the ROS-p38-p53 pathway.^80^ Lycopene is a key bioactive compound found in tomatoes. The topical application of lycopene blocks tripropylamine (TPA)-induced intracellular redox disturbances and mouse cutaneous tumors in the growth phase by speeding up nuclear factor erythroid 2-related factor 2 (Nrf2) nuclear localization. This effect may have been caused by an elevation in the levels of the autophagy protein p62, which makes it easier for Keap1 to be degraded via the autophagy-lysosomal pathway.^84^ Vanillic acid (VA), a benzoic acid derivative, ameliorates obesity and melanoma through the STAT3 pathway in mice. In a melanoma cancer-obesity comorbidity model, oral VA treatment reduced body weight, white adipose tissue weight, and tumor growth. The levels of the corresponding biomarkers confirmed the enhanced levels of autophagy.^85^ By focusing on Notch signaling, a biphenolic naturally occurring honokiol (HNK) suppresses melanoma stem cells. HNK dramatically reduces the viability, clonogenicity, proliferation, and increased autophagy of melanoma cells. Additionally, HNK substantially and dose-dependently reduces melanosphere development.^86^ Two naturally occurring compounds, isokotomolide A (Iso A) and secokotomolide A (Sec A), obtained from *Cinnamomum kotoense*, may be effective against human melanoma, particularly against B16F10, A2058, MeWo, and A375 cells. Autophagy induces and causes DNA damage and cell cycle arrest.^88^ Gracillin, another natural compound, also induces autophagy in melanoma cells. It suppresses the phosphorylation and activation of PI3K, AKT, and mTOR in melanoma cells.^89^

Similar to the HLP extract, some natural extracts regulate autophagy in skin cancer. A study found that treatment of spontaneously transformed human keratinocyte cell culture (HaCaT) with East Indian sandalwood oil (EISO), where the main component is α-santol, blocked the advancement of the cell cycle and inhibited the function of AP-1, a protein known to cause skin cancer, in a concentration-dependent manner. Additionally, EISO-treated cells exhibited extensively damaged plasma membranes, which might have caused LC3 cleavage and autophagy induction.^93^ Natto, a soy product fermented by the bacteria *Bacillus subtilis*, is used to prepare the natto freeze-drying extract (NFDE) and natto water extract (NWE). Natto is believed to play a significant role in cell death by regulating ROS and autophagy. NFDE and NWE have strong dose-dependent anti-melanoma effects but have little effect on healthy skin cells.^92^ Another medicinal plant from Belarus, *Jasione montana* L. (Campanulaceae), and its main active compound luteolin are effective against melanoma. Diethyl ether (Et_2_O) fraction of the plant extract (JM4), in which luteolin is present in high amounts, can induce autophagy in a dose-dependent manner. JM4 and its main constituents can cause cell cycle arrest and a significant reduction in the mitochondrial membrane potential.^91^ Chinese propolis (CP) has a strong antitumor effect against different malignancies. According to a previous study, the antiproliferative and anti-inflammatory properties of CP worked together to slow the growth of the human melanoma cell line A375. CP-induced autophagy was also observed in A375 cells. Interestingly, the anticancer activity of CP-treated cells decreased when autophagy was inhibited.^94^

### Natural compounds inhibiting autophagy in skin cancer

Some compounds inhibit autophagy in skin cancer, indicating a dual role of autophagy in skin cancer. Climacostol, from the protozoan *Climacostomum virens*, inhibits autophagy in melanoma B16-F10 cells by increasing p53 protein levels and activating AMPKα.^120^ Decursinol angelate is a coumarin compound that inhibits autophagy in melanoma B16-F10 cells by inhibiting ATG5, ATG7, and Beclin 1 expression and LC3-I to LC3-II conversion.^121^ Pinocembrin (5,7-dihydroxy flavanone) is a naturally occurring flavanone found in CP with diverse medicinal properties. It reduces the growth of melanoma cells (A375 and B16F10) *in vitro* in a dose-dependent manner, induces ER stress via the inositol-requiring enzyme type 1 (IRE1)/X-box binding protein 1 (Xbp1) pathway, and prompts caspase-12/caspase-4-mediated death in both cell lines. Pinocembrin also inhibits autophagy by activating the PI3K/Akt/mTOR pathway, which acts as a dual mechanism to enhance the pro-apoptotic effects of this compound.^122^

### Synthetic and semisynthetic compounds inducing autophagy in skin cancer

Ibrutinib, a Food and Drug Association (FDA)-approved synthetic medicine (also known as PCI-32765), irreversibly inhibits Bruton's tyrosine kinase.^130^ A previous study demonstrated that ibrutinib caused skin cancer cells to undergo both autophagy and apoptosis. According to western blot analysis, the ibrutinib concentration and treatment duration were associated with the development of autophagy in skin cancer cells.^95^ A novel EGCG analog 4-(S)-(2,4,6-trimethylthiobenzyl)-EGCG is a polyphenol isolated from *Camellia sinensis* L. that selectively induces ROS accumulation and autophagy in melanoma B16-F10 cells.^69^ Protoapigenone 1′-O-butyl ether, a semisynthetic p-quinol, activates autophagy in melanoma A375 cells by upregulating ROS generation and p53 levels.^65^ Dacarbazine (DTIC) is also an FDA-approved drug used in the treatment of melanoma; it induces autophagy. However, another study found that pitavastatin and DTIC were more successful than DTIC alone in treating the human melanoma cancer cell lines WM115 and A375. Pitavastatin and DTIC therapy cause synergistic cell death and G1 cell cycle arrest, and pitavastatin and DTIC therapy together activate autophagy as a component of cell death. Another drug temozolomide, which is currently in phase II clinical trial, possesses the same effect as the aforementioned two drugs do. Another combination of hydroxychloroquine and temsirolimus is responsible for inducing autophagy through the mTOR pathway and cancer cell death in human melanoma.^1^^,^^106^ A novel binuclear palladacycle complex (AJ-5) effectively induces autophagy and cell death in melanoma cancer cell lines. In a study on ME1402 and WM1158 melanoma cells, it effectively inhibited the proliferation of both cell types. AJ-5 therapy also stimulates the development of autophagosomes and increases the levels of several autophagy indicators, such as LC3-II and Beclin 1.^96^ 2-Deoxy-d-glucose, a synthetic glucose analog, induces autophagy in melanoma cells.^112^ Pyrimethamine is a synthetic therapeutic agent that induces autophagy in metastatic melanoma cells by increasing the autophagosome and LC3-II expression levels.^113^ Metformin, an antidiabetic drug, induces autophagy in melanoma cells by regulating LC3 and Beclin 1 protein levels.^114^ Ivermectin, a semisynthetic antiparasitic compound, enhances TFE3-dependent autophagy in melanoma cells via the ROS pathway.^115^ Terfenadine, an H1 histamine receptor antagonist, is a strong inducer of death in melanoma cells by manipulating Ca^2+^ homeostasis and promoting autophagy in skin melanoma cells in ROS-independent and ROS-dependent manner, according to two separate studies.^97^ Accordingly, the ROS-mitochondrial dysregulation-associated pathways are involved, at least in part, in the induction of autophagy and death by the proteasome inhibitor bortezomib in the A375 and BLM cell lines.^98^ Treatment of the antibiotic triclosan with the cell type A375 also causes mitochondrial malfunction. Triclosan induces cytotoxicity and cell death by regulating ROS signaling and promoting autophagy in melanoma A375 cells.^99^ Monobenzone treatment causes M0508A melanocytes and melanoma cells to undergo autophagocytic destruction of melanosomes and produce ROS. In addition to suppressing cellular pigment synthesis and activating dendritic cells, the vitiligo-causing substance monobenzone stimulates cytotoxic melanoma-reactive T lymphocytes, which subsequently eliminate melanoma *in vivo*.^101^ AC-1001-H3, a synthetic peptide, induces autophagy through ROS generation and elevates the levels of LC3/LC3-II and Beclin 1 in melanoma cells.^116^ Another novel substance, HA15, directly inhibits HSPA5 and increases the unfolded protein response (UPR), causing cancer cells to simultaneously die *in vitro* and *in vivo* by inducing autophagy. This substance offers strong evidence in favor of the hypothesis that ER stress inducers can be useful innovative therapeutic approaches for addressing melanoma.^103^ Lead compound Pyr-1 induces AKT-dependent autophagy in A375 cells.^117^ Under UV irradiation, Ag/ZnO nanoparticles enhance ROS generation in A375 cells, which in turn cause cytotoxicity, increased autophagic turnover, and cell death through apoptosis.^107^ Nitrogen-doped titanium dioxide nanoparticles (N-TiO_2_ NPs) activated by light increase ROS generation, which causes necroptosis and blocks autophagy due to a change in the lysosomal pH. Instead, as a pro-survival strategy in the dark, N-TiO_2_ NPs increase autophagic flow. Similar to N-P-doped carbon dots, which increase cytotoxicity and induce apoptosis, oxidative stress, and autophagy in B16-F10 cells, these particles exhibit anticancer effects.^108^^,^^109^ Notably, after therapy with BAY 87-2243, a strong inhibitor of mitochondrial complex I, tumor growth is inhibited *in vivo*. In G361 and SK-Mel 28 cells, it also promotes cell death, increases cellular ROS levels, enhances lipid peroxidation, and decreases glutathione levels, while promoting autophagosome formation and mitophagy.^102^ Rapamycin promotes autophagy in melanoma cells by blocking the mTOR pathway and increasing the protein levels of Bcl-2, Bax, and LC3-II.^118^ Lapatinib also inhibits the PI3K/Akt/mTOR signaling pathway to induce autophagy in A431 cells *in vitro*.^105^ Additionally, the synthetic flavonoid GL-V9 is developed from the naturally occurring bioactive component wogonin. In the human cutaneous SCC cell line A431 cells, GL-V9 promotes autophagy and stops the progression of chemically produced primary skin cancer in mice by blocking the Akt/mTOR pathway and triggering autophagy.^104^ Another study was conducted to determine the effect of nitidine chloride (NC) on A375 and WM35 melanoma cancer cells. It induces autophagy in melanoma cells and activates the AMPK-mTOR pathway, which is crucial for autophagy initiation. Additionally, the induction of autophagy by NC may exert a cytoprotective effect on melanoma cells because suppressing autophagy using a 3-MA or AMPK pathway inhibitor significantly enhanced apoptosis and cell death caused by NC.^87^ In this study, neratinib, an irreversible inhibitor of erythroblastic leukemia viral oncogene homolog (ErbB) 1/2/4, was investigated in uveal melanoma cells along with the histone deacetylase inhibitor entinostat. Although various mechanisms are involved, neratinib and entinostat kill cancer cells by enhancing autophagy.^100^ Delivery of an analog of photoactive nicotinamide adenine dinucleotide phosphate (NADPH) NS1 in A375, SK-Mel 28, and primary melanoma cells promotes cancer cell death by inhibiting NADPH oxidase (NOX). Although autophagy is triggered by this compound, it is only partially complete.^126^ Pheophorbide a (Pa), a derivative of chlorophyll, is a photosensitizer that exerts substantial anticancer effects in several cancer cell types. Photodynamic therapy (PDT) effectively reduces the proliferation of the two human skin cancer cell lines, A431 and G361, in a concentration-dependent manner. PDT therapy results in increased expression of the ATGs Beclin 1, LC3B, and ATG5 in A431 cells, but not in G361 cells. According to an *in vivo* investigation, Pa-PDT significantly increased autophagy and/or apoptosis in tumors that had been transplanted with A431 or G361 cells, respectively.^110^ Moreover, berberine-mediated PDT (BBR-PDT) is effective against human melanoma cells. BBR-PDT increases LC3-related autophagy. Additionally, it induces ER stress, resulting in a sharp increase in ROS. Interestingly, BBR-PDT-induced apoptosis, autophagy, and ER stress levels are decreased by C/EBP homologous protein (CHOP) production, indicating that CHOP is possibly associated with apoptosis, autophagy, and ER stress in MMCs treated with BBR-PDT.^111^ According to another study, T-type Ca^2+^ channel blockers, such as mibefradil and pimozide, could treat melanoma cells in which basal autophagy was observed. Thus, they inhibit autophagy and promote cell death in malignant melanoma cells.^128^

### Synthetic and semisynthetic compounds inhibiting autophagy in skin cancer

Sorafenib, an FDA-approved drug and multi-kinase inhibitor, inhibits autophagy during melanoma treatment.^1^ Natural compound α-mangostin enhances the activity of sorafenib and synergistic effects in the suppression of autophagy by inhibiting Akt, and ERK is observed in neuroblastoma RAS viral oncogene homolog-mutant melanoma cells.^129^ Hydroxychloroquine is a therapeutic compound that inhibits autophagy by blocking autophagosome–lysosome fusion in melanoma cells.^131^ Chloroquine also acts as a lysosomal autophagy inhibitor and kills melanoma cells in an autophagy-independent manner. In addition to chloroquine, the lysosomal autophagy inhibitors bafilomycin A1 and ammonium chloride possess the same effect in B16-F0 cells exposed to high levels of oxidative stress and autophagy-independent mitochondrial depolarization, which together cause caspase-mediated death.^124^ 2-Phenylethynesulfonamide in combination with NVP-AUY922 also inhibits autophagy by increasing glutathione levels in melanoma cells.^43^ Additionally, esomeprazole induces ROS-dependent cell death in Me30966, Mel501, and WM793 cell lines by inhibiting autophagy, one of the adverse modes of action of the drug. Through a caspase-dependent mechanism, it kills melanoma cells while decreasing autophagic flow.^127^ 3-MA is a known autophagy inhibitor that has a synergistic effect against SSC in combination with 5-fluorouracil. This combination inhibits the proliferation and metastasis of cancer cells. LC3 expression is negatively associated with Bcl-2 expression and/or survival, confirming autophagy in SCC.^125^

## Limitations and future prospects

In this review, we systematically examined the involvement of autophagy in skin cancer and evaluated the efficacy of autophagy modulators as potential treatments. However, this study has some limitations. First, the precise role of autophagy in cancer remains debatable, necessitating further experiments to comprehensively elucidate its functions and signaling pathways in the context of skin cancer. Second, considering that autophagy is a ubiquitous metabolic process in human physiology, the safety profile of autophagy modulators for the treatment of skin cancer warrants careful investigation. Furthermore, although this study explored promising autophagy modulators identified *in vitro* and *in vivo* studies, their effectiveness must be rigorously validated in human models for translation.

## Conclusion

Skin cancer remains one of the major health problems to be solved, and alternative targeted therapies have been explored owing to their optimum safety, effectiveness, and potential. Autophagy is considered one of these factors as it plays a dual role in skin cancer. Although the exact role of autophagy in skin cancer remains controversial, it plays an important role throughout cancer development, making it a crucial target for understanding and effectively treating skin cancer. In the initial stages, autophagy suppresses tumor progression and prevents the progression of cancer to advanced stages. However, when cancer reaches advanced stages, autophagy helps cancer cells survive and grow exponentially. Thus, autophagy inducers and inhibitors, collectively referred to as autophagy modulators, can be used to inhibit skin cancer progression and effectively reduce cancer cell growth. Based on our current understanding, this review provides the first comprehensive examination of a clinical approach for targeting autophagy and the role of autophagy modulators in the treatment of skin cancer. Based on our findings, both the promotion and suppression of autophagy are beneficial in addressing skin cancer. Particularly, our study delineated the involvement of autophagy-related molecular pathways, including the AMPK and mTOR pathways, in the regulation of skin cancer using natural, semisynthetic, and synthetic molecules within the human body. Further investigations are necessary to fully understand the role of autophagy at different stages of skin cancer and the effectiveness of autophagy modulators in treating skin cancer.

## Funding

None.

## Authors contribution

Md. Liakot Ali: conceptualization, writing – original draft, data extraction, and data analysis; Amdad Hossain Roky: writing – original draft and data extraction; S.M. Asadul Karim Azad: writing – original draft; Abdul Halim Shaikat: writing – original draft; Jannatul Naima Meem: writing – original draft; Emtiajul Hoque: writing – original draft; Abu Mohammed Fuad Ahasan: writing – original draft; Mohammed Murshedul Islam: writing – original draft; Md. Saifur Rahaman Arif: writing – original draft; Md. Saqline Mostaq: writing – original draft; Md. Zihad Mahmud: writing – original draft; Mohammad Nurul Amin: project administration; Md. Ashiq Mahmud: conceptualization, supervision, and manuscript revision. All authors reviewed and approved the submission of the final version of the manuscript.

## Ethics statement

None.

## Data availability statement

The datasets used in this study can be obtained from the corresponding author upon reasonable request.

## Conflict of interest

The authors declare that they have no known competing financial interests or personal relationships that could have appeared to influence the work reported in this paper.

## Acknowledgments

None.
